# TH588 and Low-Dose Nocodazole Impair Chromosome Congression by Suppressing Microtubule Turnover within the Mitotic Spindle

**DOI:** 10.3390/cancers13235995

**Published:** 2021-11-29

**Authors:** Girish Rajendraprasad, Susana Eibes, Claudia Guasch Boldú, Marin Barisic

**Affiliations:** 1 Cell Division and Cytoskeleton, Danish Cancer Society Research Center, 2100 Copenhagen, Denmark; girish@cancer.dk (G.R.); susana@cancer.dk (S.E.); guasch.claudia@gmail.com (C.G.B.); 2 Department of Cellular and Molecular Medicine, Faculty of Health Sciences, University of Copenhagen, 2200 Copenhagen, Denmark

**Keywords:** microtubule dynamics, microtubule-targeting agents, cancer therapy, mitotic spindle, cell division

## Abstract

**Simple Summary:**

A promising anti-cancer compound TH588 has been recently identified as a microtubule-targeting agent that inhibits tubulin polymerization in vitro and interferes with microtubule dynamics in interphase cells. Although it was shown to arrest cells in mitosis, its effect on microtubule dynamics in dividing cells remained unknown. By analyzing microtubule dynamics in living cells treated with either TH588 or low-dose nocodazole, we revealed that both of these drugs stabilize microtubules within the mitotic spindle, leading to premature formation of kinetochore-microtubule end-on attachments on uncongressed chromosomes. This causes mitotic arrest, ultimately resulting in cell death or cell division with uncongressed chromosomes. Both of these cell fates could contribute to the selective effect associated with the activity of TH588 in cancer cells.

**Abstract:**

Microtubule-targeting agents (MTAs) have been used for decades to treat different hematologic and solid cancers. The mode of action of these drugs mainly relies on their ability to bind tubulin subunits and/or microtubules and interfere with microtubule dynamics. In addition to its MTH1-inhibiting activity, TH588 has been recently identified as an MTA, whose anticancer properties were shown to largely depend on its microtubule-targeting ability. Although TH588 inhibited tubulin polymerization in vitro and reduced microtubule plus-end mobility in interphase cells, its effect on microtubule dynamics within the mitotic spindle of dividing cells remained unknown. Here, we performed an in-depth analysis of the impact of TH588 on spindle-associated microtubules and compared it to the effect of low-dose nocodazole. We show that both treatments reduce microtubule turnover within the mitotic spindle. This microtubule-stabilizing effect leads to premature formation of kinetochore-microtubule end-on attachments on uncongressed chromosomes, which consequently cannot be transported to the cell equator, thereby delaying cell division and leading to cell death or division with uncongressed chromosomes.

## 1. Introduction

Microtubules (MTs) are filamentous tubular structures, typically consisting of 13 laterally associated protofilaments that are built from α- and β-tubulin heterodimers. MTs are highly dynamic, owing to the so-called “dynamic instability” [1,2,3,4,5] that is characterized by constant switching between persistent phases of elongation and shortening. The switch from shortening to elongation is called “rescue” and depends on the formation of a protective “cap” resulting from incorporation of GTP-tubulin into the growing MT end. Within the MT lattice, GTP-tubulin is transformed into GDP-tubulin via hydrolysis. The switch from elongation to shortening is known as “catastrophe” and is characterized by the loss of GTP-tubulin cap that leads to dissociation of GDP-tubulin from the MT [5,6,7].

MTs are essential for several key cellular roles. In interphase, they are required for intracellular trafficking and maintenance of cell shape and migration. In mitosis they build the mitotic spindle and connect it to the cell cortex to promote proper orientation of cell division, as well as they bind to the kinetochores at the centromeric region of chromosomes and drive chromosome segregation. Interference with these critical functions can lead to cell death, making MTs an important anticancer drug target. Indeed, microtubule-targeting agents (MTAs) have been used for decades to treat different hematologic and solid cancers. The mode of action of these drugs mainly relies on their ability to bind tubulin subunits and/or MTs and interfere with MT dynamics. Specific MTAs mainly belong to one of the two categories: MT-stabilizing agents (e.g., taxanes) that promote tubulin polymerization and/or prevent MT depolymerization, and MT-destabilizing agents (e.g., vinca alkaloids) that prevent tubulin polymerization and/or promote MT depolymerization. MTAs can induce cancer cell death by disrupting the mitotic spindle, thereby leading to mitotic arrest-associated cell death [8] or to mitotic slippage followed by the cell death in subsequent cell cycles [9,10,11]. In addition, MTAs can induce cell death of non-dividing interphase cells by affecting intracellular transport, cell signaling, and motility [12].

However, the therapeutic efficacy of these compounds is limited by two main drawbacks. First, since MTs are essential for intracellular transport in neurons, these agents can cause severe side-effects, such as neuropathies and neurotoxicity in patients. Second, another enormous clinical challenge is caused by the development of drug resistance against MTAs. Thus, novel classes of MTAs that could overcome these problems are highly needed. One such molecule that showed great potential in preclinical studies is TH588 and its derivatives [13,14,15,16,17,18]. Compared to primary or immortalized, non-transformed cells, TH588 displayed selective toxicity against various human cancer cell lines and it effectively reduced tumor growth in mice [13]. It was originally identified as an inhibitor of the mut-T homolog 1 (MTH1, also known as NUDT1), a hydrolase that prevents oxidized dNTPs to be incorporated into DNA [19]. Therefore, its anticancer effect was initially proposed to originate from a bigger dependency of the cancer cells to reduce oxidative DNA damage via MTH1 activity [13,20]. However, in addition to its MTH1-inhibiting activity, TH588 has been recently identified as an MTA, and the anticancer properties of this dual inhibitor were shown to largely depend on its ability to target MTs [15,21,22,23].

In vitro, TH588 inhibited tubulin polymerization in a dose-dependent manner [15,22] and reduced MT plus-end mobility in interphase cells [21]. By interfering with the functionality of mitotic spindle, TH588 induced mitotic arrest that was sporadically followed by mitotic slippage [21]. However, how MT dynamics within the mitotic spindle of dividing cells are affected by TH588 remains unknown.

Recently resolved X-ray crystal structure of the α/β-tubulin:TH588 complex revealed that TH588 binds to the colchicine binding site of β-tubulin, thereby blocking tubulin polymerization and MT assembly. Moreover, mutation of the TH588-binding site in β-tubulin decreased the TH588-mediated cytotoxicity, highlighting the importance of MT-targeting for TH588-induced cell death [22]. Interestingly, the resolved structure also revealed a close overlap in binding to the colchicine site between TH588 and nocodazole, a known MT-depolymerizing agent, suggesting that both TH588 and nocodazole prevent tubulin straightening by perturbing the compaction within this specific site in β-tubulin [22].

Although structural and in vitro data would suggest that TH588 has a similar MT-depolymerizing mode of action as nocodazole, TH588 reduced the MT plus-end mobility and slowed down MT dynamics in interphase cells, indicating its MT-stabilizing effect on non-dividing cells. In order to better understand the impact of TH588 on MT dynamics and how it affects mitosis, in this study we performed an in-depth analysis of the effects of TH588 and nocodazole on MT dynamics within the mitotic spindles in dividing human cancer cells. We show that, both TH588 and low concentration of nocodazole reduce MT turnover within the mitotic spindle. This reduction of MT dynamics leads to premature stabilization of kinetochore-MT end-on attachments, finally inducing severe chromosome congression problems that leading to mitotic arrest, which is to a certain extent followed by cell division with unresolved erroneous kinetochore-MT attachments.

## 2. Materials and Methods

### 2.1. Tubulin Polymerization Assay

Assembly competent tubulin was purified from porcine brain as described before [24]. Turbidity-based MT polymerization assay was performed as described before [25,26]. Briefly, indicated concentrations of the drugs or DMSO (vehicle control) were mixed with free tubulin (2 mg/mL final concentration) and MT assembly was induced by the addition of 1 mM GTP and 10% glycerol in BRB80 buffer (80 mM PIPES, pH 6.8, 1 mM MgCl_2_, and 1 mM EGTA) at 37 °C. MT polymerization was monitored by measuring the change in absorbance (340 nm) using SpectraMax iD3 (Molecular Devices, San Jose, CA, USA) microplate reader in 30 s intervals over 60 min.

### 2.2. Cell Culture

U2OS and HeLa cell lines were grown in DMEM (Gibco) and RPE-1 in DMEM/F12 media (Gibco) supplemented with 10% FBS (Invitrogen, Waltham, MA, USA) at 37 °C with 5% CO_2_.

### 2.3. Immunofluorescence Imaging

Antibodies used in this study are mouse anti-alpha-tubulin (1:2000; Sigma-Aldrich–T5168; B-5-1-2, St. Louis, MO, USA), rabbit anti-centrin (1:2000; gift from I. Cheeseman, Whitehead Institute for Biomedical Research, Cambridge, MA, USA), rabbit anti-ZW10 (1:50; ab21582, Abcam, Cambridge, UK), guinea pig anti-CENP-C (1:2000; PD030, MBL International, Woburn, MA, USA), Alexa Fluor conjugated secondary antibodies (1:1000, Thermo Fisher Scientific, Waltham, MA, USA), and DAPI (1 µg/mL, Sigma-Aldrich, St. Louis, MO, USA) as DNA counterstain.

For MT regrowth assays, MTs were first depolymerized by incubating U2OS cells on ice in presence of DMSO, 5 µM TH588 (MedChemExpress, Monmouth Junction, NJ, USA), or 100 nM nocodazole (Sigma-Aldrich, St. Louis, MO, USA) for 30 min. To induce MT regrowth, cells were incubated with warm DMEM media containing the specified drugs. Cells were immediately fixed with ice-cold methanol at indicated time points and MTs and centrin were stained using anti-alpha-tubulin and anti-centrin antibodies, respectively. Images were acquired using Zeiss AxioObserver Z1 wide-field microscope (63× Plan-Apochromatic oil differential interference contrast objective lens, 1.4 NA) equipped with Metal halide arc lamp and Axiocam 702 mono CMOS camera and Zen 3.0 blue edition software (Carl Zeiss, Inc., Oberkochen, Germany). Representative images were acquired using LSM700 confocal microscope (Carl Zeiss Inc., Oberkochen, Germany) mounted on a Zeiss-Axio imager Z1 equipped with plan-apochromat 63×/1.40 oil DIC M27 objective (Carl Zeiss, Inc., Oberkochen, Germany) and Zen 2008 software (Carl Zeiss, Inc., Oberkochen, Germany). For quantification of MT nucleation, alpha-tubulin intensity at 1 min time-point was selected by a circular ROI covering the centrosome, followed by subtraction of the cytoplasmic background. The resulting intensity values were normalized to the median of control. Centrin was used to define the centrosomal position.

For quantification of MT populations in mitotic cells, U2OS parental cells were arrested in metaphase using APC/C inhibitors Apcin (Sigma-Aldrich) (20 µM) and proTAME (BostonBiochem, Cambridge, MA, USA) (10 µM) for 2 h followed by treatment with indicated drugs or DMSO for 1 h. Following the treatment, the cells were fixed with 4% paraformaldehyde in PHEM buffer for 20 min at 37 °C as described before [27,28] and MTs were stained using anti-alpha-tubulin antibody. Images were acquired using a Plan-Apochromat 63×/1.4 NA oil objective with differential interference contrast mounted on an inverted Zeiss Axio Observer Z1 microscope (Marianas Imaging Workstation, 3i-Intelligent Imaging Innovations, Inc., Denver, CO, USA) equipped with an iXon Ultra 888 EM-CCD camera (Andor Technology, Belfast, UK). Astral and spindle MT intensities were quantified using ImageJ (National Institute of Health, Bethesda, MD, USA) as described before [26].

To study the localization pattern of ZW10 and kinetochore-MT attachments, U2OS cells were treated with indicated drugs for 1 h, followed by fixation using ice-cold methanol and staining using target specific primary antibodies and Alexa Fluor conjugated secondary antibodies. Images were acquired using LSM700 confocal microscope described above. Line intensity profiles were obtained using plot profile function in ImageJ.

### 2.4. Live Cell Imaging

Live-cell time-lapse imaging was performed as described before [29,30]. Briefly, cells were cultured in 35 mm glass-bottomed dishes (14 mm, No. 1.5, MatTek Corporation, Ashland, MA, USA) and imaging was performed in an environment controlled chamber (37 °C with controlled humidity and 5% CO_2_ supply), using a Plan-Apochromat DIC 63×/1.4 NA oil objective mounted on an inverted Zeiss Axio Observer Z1 microscope (Marianas Imaging Workstation from 3i-Intelligent Imaging and Innovations Inc., Denver, CO, USA), equipped with a CSU-X1 spinning-disk confocal head (Yokogawa Corporation of America, Sugar Land, TX, USA) and four laser lines (405 nm, 488 nm, 561 nm and 640 nm). Images were acquired using an iXon Ultra 888 EM-CCD camera (Andor Technology).

To observe MT dynamics during interphase, U2OS EB1-GFP cells [31] (gift from P. Draber, Institute of Molecular Genetics of the Czech Academy of Sciences-IMG ASCR, Prague, Czech Republic) were treated with indicated drugs 15 min before imaging. To study MT dynamics during mitosis, cells were arrested in metaphase using anaphase-promoting complex/cyclosome (APC/C) inhibitors Apcin (20 µM) and a cell permeable tosyl-L-arginine methyl ester (proTAME) (10 µM) for 2 h before the addition of the drugs. DNA was labelled by adding 20 nM SiR-DNA (Spirochrome) 1 h prior to live-cell imaging. Cells were imaged at 2 frames per second rate for 1–2 min. About 2–5 EB1 comets per cell were tracked manually using ImageJ.

To study the mitotic effects of TH588 and nocodazole, U2OS cells stably expressing H2B-GFP/mCherry-α tubulin [32], U2OS cells stably expressing PA-GFP/mCherry-α-tubulin/CENP-A-GFP [33], HeLa cells stably expressing H2B-mCherry [34] (gift from D. Gerlich, Institute of Molecular Biotechnology of the Austrian Academy of Sciences-IMBA, Vienna, Austria) with MTs stained using 10 nM SiR-tubulin (Cytoskeleton Inc., Denver, CO, USA), and RPE-1 cells stably expressing H2B-GFP/RFP-α tubulin (gift from H. Maiato, Institute for Research and Innovation in Health–i3S, Porto, Portugal) were treated with the drugs for 15 min and imaged using fifteen 1 μm-separated z-planes collected every 2 min.

MT flux and turnover rates in mitotic spindles were imaged and quantified using U2OS-PA-GFP/mCherry-α-tubulin cells [35] (gift from R. Medema, Netherlands Cancer Institute-NKI, Amsterdam, Netherlands) as described before [28,29]. The cells were arrested in metaphase using 5 µM MG132 for 1 h followed by treatment with indicated drugs for 15 min.

### 2.5. Image Processing and Statistical Analysis

All graphs and statistical analysis were generated in GraphPad Prism 8.0. Data points were tested for normality using D’Agostino & Pearson test. Accordingly, statistical significance was determined by Student’s *t*-test (unpaired, two-tailed; normal distribution) or Mann-Whitney *U*-test (unpaired, two-tailed; no normal distribution). F-test was used to compare variances and Welch’s correction was employed when variances were not equal. Details of the statistical significance and n values for each conditions can be found in the figures and figure legends.

## 3. Results

### 3.1. TH588 Disrupts In Vitro MT Polymerization and in Cellulo MT Nucleation and Reduces MT Dynamics in Interphase Cells

**Figure 1:**
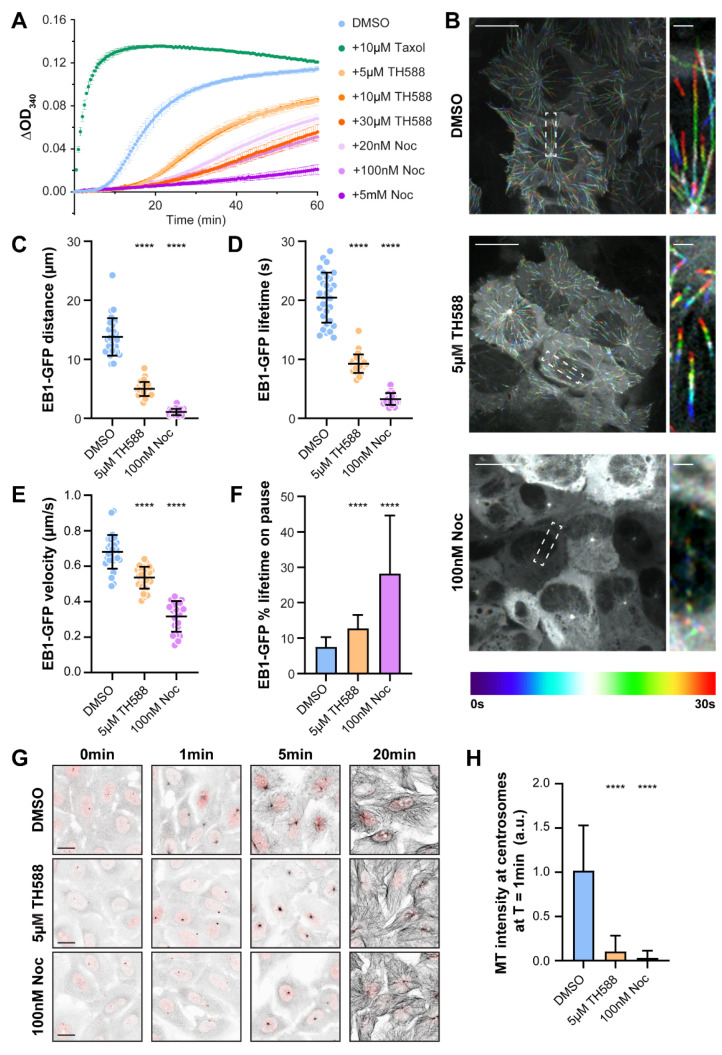
TH588 disrupts in vitro MT polymerization and in cellulo MT nucleation and reduces MT dynamics in interphase cells. (**A**) Turbidimetry-based in vitro tubulin polymerization assay showing a concentration-dependent effect of TH588 and nocodazole on polymerization dynamics. DMSO (vehicle) and Taxol serve as controls for the experimental setup. The mean and SEM are shown from three to four independent experiments. (**B**) Representative color-coded temporal projection of U2OS EB1-GFP cells undergoing indicated treatment. Scale bar 20 µm. Zoomed insets show the dynamics of individual EB1-GFP comets. Scale bar 2 µm. (**C**–**F**) Quantification of MT dynamics acquired from manual tracking of EB1-GFP comets as an indicator for MT growth. The mean and SD are plotted from three independent experiments (n = 30 cells for DMSO and 5 µM TH588, 21 cells for 100 nM nocodazole). (**G**) Representative scanning confocal images of MT regrowth assay in U2OS cells with indicated treatment. Following cold-induced depolymerization, MTs were re-polymerized by addition of warm media, fixed at the indicated time points and stained for α-tubulin (black) and centrin (red). Scale bar 20 µm. (**H**) Quantification of centrosomal nucleation measured as MT intensity around centrosome following indicated treatments. Bar graph is plotted with SD (n = 73 cells for DMSO, 69 cells for 5 µM TH588, and 71 cells for 100 nM nocodazole). **** *p* < 0.0001.

In order to test the direct effect of TH588 on MTs, we first performed a turbidity-based in vitro MT polymerization assay in the presence of TH588 at varying concentrations (Figure 1A). In agreement with previous reports [15,22], our data show a strong effect of TH588 on MT dynamics, wherein it severely affected MT polymerization in a concentration-dependent manner.

To analyze the effect of TH588 on MT dynamics in cellulo, we performed spinning-disk confocal microscopy-based live-cell imaging of human osteosarcoma U2OS cells stably expressing EB1-GFP, a MT-plus-end tracking protein that localizes to the tips of growing MTs. Because structural [22] and in vitro [15,22] data suggest a similar MT-depolymerizing mode of action between TH588 and nocodazole, we compared the effect of these two drugs on MT dynamics. Tracking of EB1-GFP comets in interphase cells upon individual treatments with either TH588 or low-dose nocodazole revealed a drastic effect of both drugs on the growth of MTs, represented by the distance traversed by EB1-GFP (4.98 ± 1.18 µm with 5 µM TH588 and 1.07 ± 0.53 µm with 100 nM nocodazole, compared to 13.81 ± 3.18 µm in DMSO control) and its lifetime (9.27 ± 1.56 s with 5 µM TH588 and 3.27 ± 0.99 s with 100 nM nocodazole, compared to 20.46 ± 4.25 s in DMSO control) (Figure 1B–D, Video S1). In addition, we also observed a reduction in the EB1-GFP comet velocity (0.53 ± 0.06 µm/s with 5 µM TH588 and 0.32 ± 0.09 µm/s with 100 nM nocodazole, compared to 0.68 ± 0.1 µm/s in DMSO control), coupled with spending a significantly higher percentage of lifetime in the paused state (12.77 ± 3.8% with 5 µM TH588 and 28.19 ± 16.44% with 100 nM nocodazole, compared to 7.57 ± 2.73% in DMSO control) (Figure 1E,F, Video S1).

Taken together, these data suggest that both TH588 and low dose of nocodazole reduce MT dynamics in cellulo, showing the features characteristic for the MT dynamics-suppressing MTAs. This is in agreement with earlier observation that TH588 reduces interphase MT dynamics in H460 human large-cell lung carcinoma cells [21], as well as with the fact that, besides being a well-established MT-depolymerizing agent, nocodazole at low concentrations has a MT-stabilizing effect [36,37].

Next, we tested the impact of TH588 on MT nucleation. Similar to low-dose nocodazole, TH588 substantially reduced centrosome-mediated re-nucleation after cold-induced depolymerization of MTs (Figure 1G,H). Notably, 20 min after re-initiation of MT nucleation, the MT network was restored to control levels in both TH588 and low-dose nocodazole-treated cells (Figure 1G).

### 3.2. TH588 Induces Severe Chromosome Congression Problems That Lead to Mitotic Arrest, Followed by the Cell Death or Erroneous Cell Division

TH588 impaired chromosome congression to the spindle equator in H460 cells, inducing mitotic arrest [21]. To examine whether TH588 has the same impact on mitosis in two other cancer cell lines (U2OS and HeLa), as well as in non-tumor, hTERT-immortalized, human retinal pigment epithelial (RPE-1) cells, we performed live cell imaging and monitored mitotic duration, chromosome movement, and cell fate.

**Figure 2:**
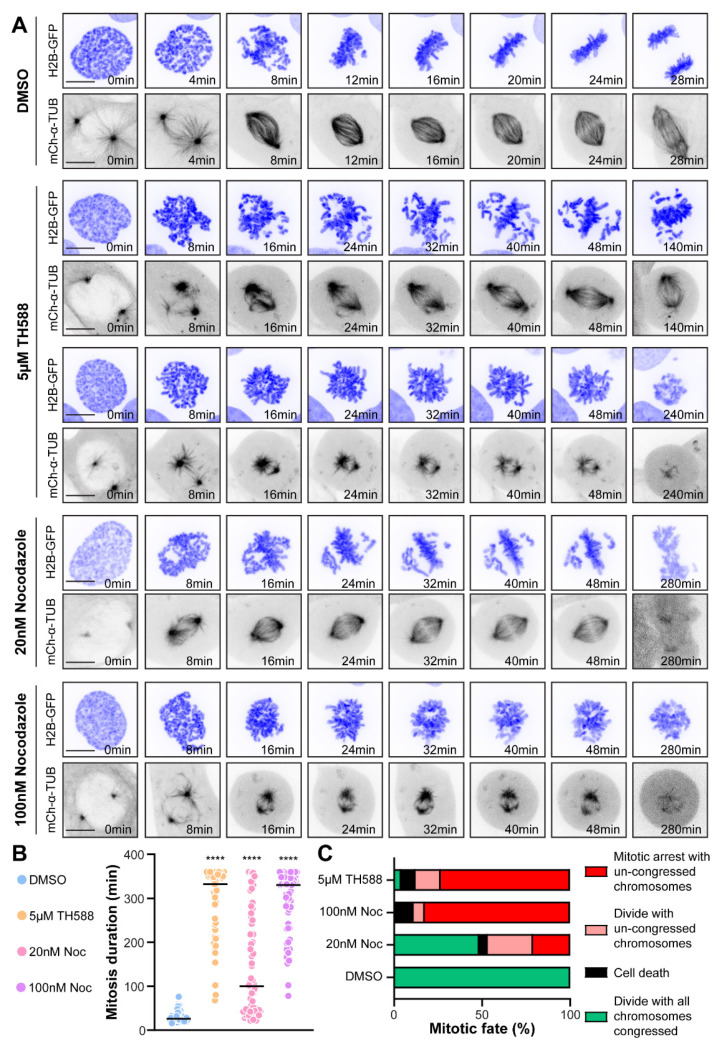
TH588 induces severe chromosome congression problems that lead to mitotic arrest, followed by the cell death or erroneous cell division. (**A**) Representative spinning disk confocal time-series of mitosis in U2OS cells stably expressing H2B-GFP/mCherry-α-tubulin undergoing indicated treatments. Scale bar 10 µm. (**B**) Quantification of duration of mitosis in U2OS cells undergoing indicated treatments. Scatter plot graphs with median are plotted from three independent experiments (n = 56 cells for DMSO, 49 cells for 5µM TH588, 63 cells for 100 nM nocodazole, and 65 cells for 20 nM nocodazole). (**C**) Quantification of the fate of mitotic cells from three independent experiments. **** *p* < 0.0001.

First, we compared the effects of TH588 and low-dose nocodazole treatments in U2OS cells stably expressing histone 2B (H2B)-GFP and mCherry-α-tubulin. Whereas control cells formed a proper bipolar spindle, congressed the chromosomes to the metaphase plate and divided normally (Video S2), both TH588 and nocodazole treatments drastically affected mitotic progression. About 5 µM TH588 induced severe chromosome congression problems, represented by multiple chromosomes getting trapped around the spindle poles, unable to reach the metaphase plate (Figure 2A,B, Video S3). This effect of TH588 on chromosome congression and occasional spindle-collapsing strongly resembled the mitotic phenotype observed upon treating the U2OS cells with 20 nM and 100 nM nocodazole (Figure 2A,B, Video S4). We further analyzed the fate of mitotic cells during 6 h after addition of 5 µM TH588. While all DMSO-treated control cells divided normally, we observed that 73.47% of the treated cells were arrested in mitosis due to the problems in chromosome congression, whereas 14.26% of cells entered anaphase with uncongressed chromosomes and 8.16% of cells died (Figure 2C). Similar cell fates were observed upon the treatment with nocodazole, where 20 nM concentration showed a milder phenotype (20.97% of cells arrested in mitosis with uncongressed chromosomes, 25.81% of cells divided with uncongressed chromosomes and 4.84% of cells died), compared to the cells treated with 100 nM (82.54% of cells arrested in mitosis with uncongressed chromosomes, 6.35% of cells divided with uncongressed chromosomes and 11.12% of cells died) (Figure 2C).

A very strong mitotic effect was also observed in HeLa cells stably expressing H2B-mCherry treated with either TH588 or 100 nM nocodazole. During 6 h of imaging after the addition of each drug, 100% of HeLa cells stayed arrested in mitosis with uncongressed chromosomes (Figure S1A–C, Video S5). Interestingly, although TH588-treated RPE-1 cells stably expressing H2B-GFP and RFP-α-tubulin showed similar problems in chromosome congression (68% of cells arrested in mitosis with uncongressed chromosomes, 8% of cells divided with uncongressed chromosomes and 4% of cells died) during 6 h of imaging after the addition of drug, a larger fraction of RPE-1 cells managed to accurately congress all chromosomes and divide normally (20%), compared to U2OS and HeLa cells, as well as to nocodazole-treated RPE-1 cells (100% of cells arrested in mitosis with uncongressed chromosomes) (Figure S2A–C, Video S6).

### 3.3. Similar to Low-Dose Nocodazole, TH588 Disrupts a More Dynamic Array of Astral MTs, While Stabilizing Longer-Lived Kinetochore-MTs

**Figure 3:**
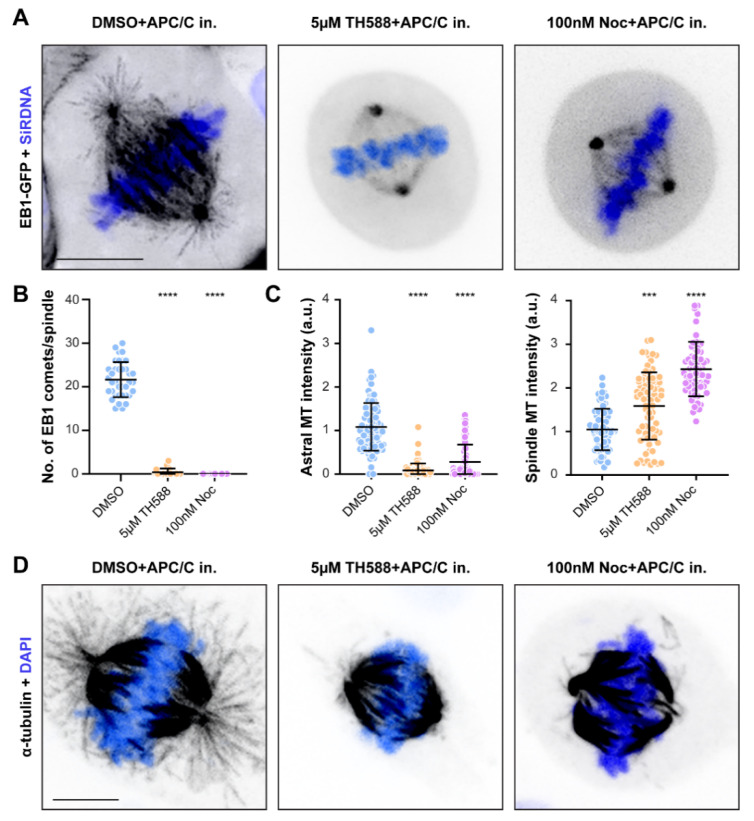
Both TH588 and low-dose nocodazole disrupt a more dynamic array of astral MTs, while stabilizing longer-lived kinetochore-MTs. (**A**) Representative temporal projection of metaphase arrested mitotic spindles of U2OS EB1-GFP cells undergoing specified treatment. DNA counterstained with SiR-DNA shown in blue. Scale bar 10 µm. Scatter plots with mean and SD are plotted from three independent experiments. (**B**) Quantification of the number of EB1 comets per mitotic spindle in metaphase arrested cells (n = 37 cells for DMSO, 37 cells for 5 µM TH588, and 36 cells for 100 nM nocodazole). (**C**) Immunofluorescence-based quantification of astral and spindle MT population intensities in metaphase-arrested mitotic cells undergoing indicated treatments. (n = 76 cells for DMSO, 80 cells for 5 µM TH588, and 66 cells for 100 nM nocodazole). (**D**) Representative images of metaphase-arrested U2OS mitotic spindles immunostained with α-tubulin antibody undergoing indicated treatments (quantified in **C**). DNA was counterstained with DAPI (blue). Scale bar 10 µm. *** *p* < 0.001, **** *p* < 0.0001.

Although TH588 reduced MT plus-end mobility in interphase cells, its effect on MT dynamics within the mitotic spindle of dividing cells remains elusive. To address this, we performed live-cell imaging of U2OS cells stably expressing EB1-GFP and monitored the impact of TH588 and low-dose nocodazole on highly dynamic astral MTs. In order to bypass the TH588 and nocodazole-associated severe congression problems that could otherwise interfere with astral MTs, we used anaphase promoting complex/cyclosome (APC/C) inhibitors to arrest cells in metaphase prior to the drug treatments. By quantifying the number of EB1 comets, we observed a severe reduction in astral MT dynamics upon the treatment with TH588 or low-dose nocodazole (Figure 3A,B, Video S7). In agreement with these data, the immunostaining of metaphase-arrested cells showed a near-total loss of astral MT population upon application of either of these two drugs (Figure 3C,D). Interestingly, although TH588 and low-dose nocodazole fully disrupted astral MTs, the quantification of fluorescence intensity upon immunostaining revealed a significant increase in the spindle MTs signal (Figure 3C,D), suggesting that TH588 and low-dose nocodazole have a MT-stabilizing effect on longer-lived MTs.

### 3.4. TH588 and Low Concentration of Nocodazole Similarly Reduce MT Turnover within the Mitotic Spindle

**Figure 4:**
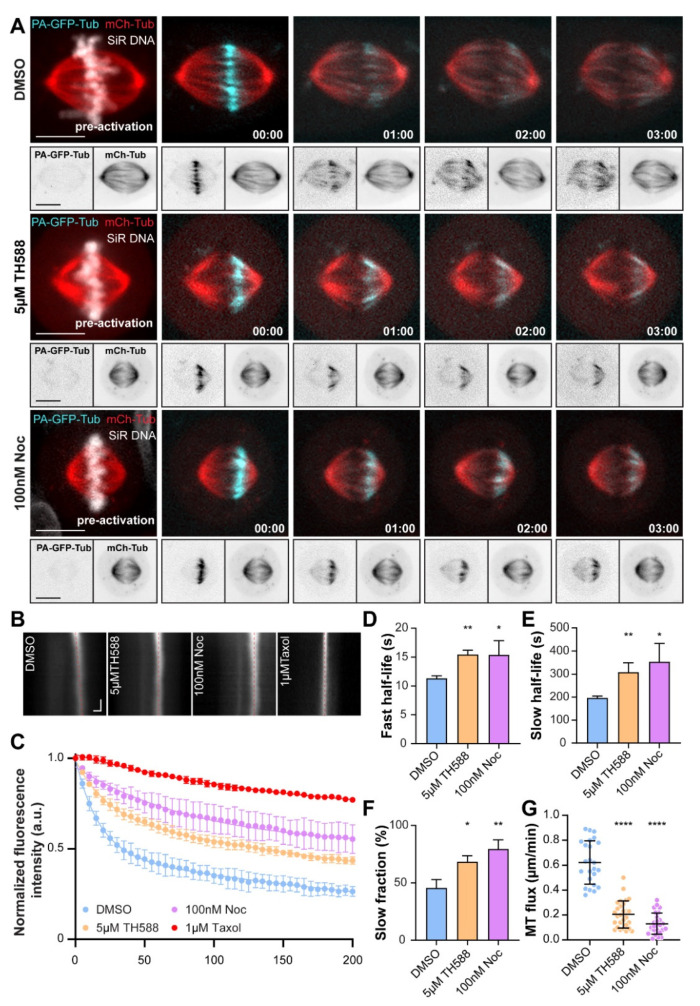
TH588 and low concentration of nocodazole similarly reduce MT turnover within the mitotic spindle. (**A**) Representative spinning-disk confocal time-lapse images showing MT turnover in U2OS-PA-GFP/mCherry α-tubulin cells undergoing indicated treatments. Metaphase-arrested one half-spindle MTs were photoactivated and tracked over time. DNA counterstained using SiR-DNA is shown in white in the first merged image of the time series while photoactivated MTs are in cyan and mCherry-α-tubulin in red. Bottom panels show inverted individual channels of PA-GFP and mCherry-α-tubulin channels. Scale bar 10 µm. Time, min:s. (**B**) Representative sum-projected kymograph profiles of photoactivated MTs in metaphase-arrested mitotic spindles of U2OS-PA-GFP/m-cherry α-tubulin cells undergoing indicated treatments (red dashed lines highlight the presence or absence of MT poleward flux). Scale bars, 2 µm (horizontal) and 30 s (vertical). (**C**) Exponential decay of normalized, photobleaching corrected fluorescence intensity of photoactivated α-tubulin over time. Data represent mean ± SEM from three independent experiments (n = 25 cells for DMSO, 31 cells for 5 µM TH588, and 22 cells for 100 nM nocodazole). Total of 1 µM taxol treatment profile serves as a control for bleaching-correction. (**D**–**F**) Fast and slow half-lives representing non-kinetochore MTs and kinetochore MTs respectively, and fast fraction obtained by fitting the data with a double exponential function. Mean ± SD are shown. (**G**) MT flux rates of metaphase arrested mitotic spindles undergoing indicated treatments shown as scatter plot with their mean and SD (n = 24 cells for DMSO, 28 cells for 5 µM TH588, and 26 cells for 100 nM nocodazole). * *p* < 0.05, ** *p* < 0.01, **** *p* < 0.0001.

In order to perform an in-depth analysis of MT dynamics within the mitotic spindle of TH588- and nocodazole-treated cells, we quantified MT turnover and poleward flux rates in metaphase-arrested U2OS cells stably expressing PA-GFP/mCherry-α-tubulin. MT turnover was measured by quantifying the fluorescence dissipation after photoactivation (FDAPA), while MT flux was determined as the velocity with which the photoactivated region traversed toward the spindle pole. FDAPA analysis revealed decreased spindle MT turnover both in TH588- and low-dose nocodazole-treated cells. This was represented by increase in both fast (15.44 ± 0.77 s with 5 µM TH588 and 15.39 ± 2.42 s with 100 nM nocodazole, compared to 11.34 ± 0.43 s in DMSO control) and slow fraction of MT half-life (308.2 ± 40.84 s with 5 µM TH588 and 352.8 ± 79.91 s with 100 nM nocodazole, compared to 196.4 ± 7.8 s in DMSO control), indicating stabilization of both non-kinetochore- (interpolar) and kinetochore-MTs (also known as k-fibers) (Figure 4A–E, Video S8). Moreover, we also observed an increase in slow fraction of MT half-life, corresponding to longer-lived kinetochore-MTs (68.36 ± 5.2% with 5 µM TH588 and 79.57 ± 7.92% with 100 nM nocodazole, compared to 45.57 ± 7.35% in DMSO control), additionally highlighting the MT-stabilizing effect of TH588- and low concentration of nocodazole (Figure 4F). Correspondingly, both TH588 and low-dose nocodazole caused a significant reduction in MT flux rates (0.21 ± 0.11 µm/s with 5 µM TH588 and 0.13 ± 0.09 µm/s with 100 nM nocodazole, compared to 0.62 ± 0.17 µm/s in DMSO control), characteristic for less dynamic, more stable MTs (Figure 4G, Video S8).

Taken together, these results demonstrate that TH588 and low-dose nocodazole stabilize spindle MTs by suppressing their dynamics.

### 3.5. TH588 Triggers Mitotic Arrest and Erroneous Cell Divisions via Premature Stabilization of Kinetochore-MT End-On Attachments

**Figure 5:**
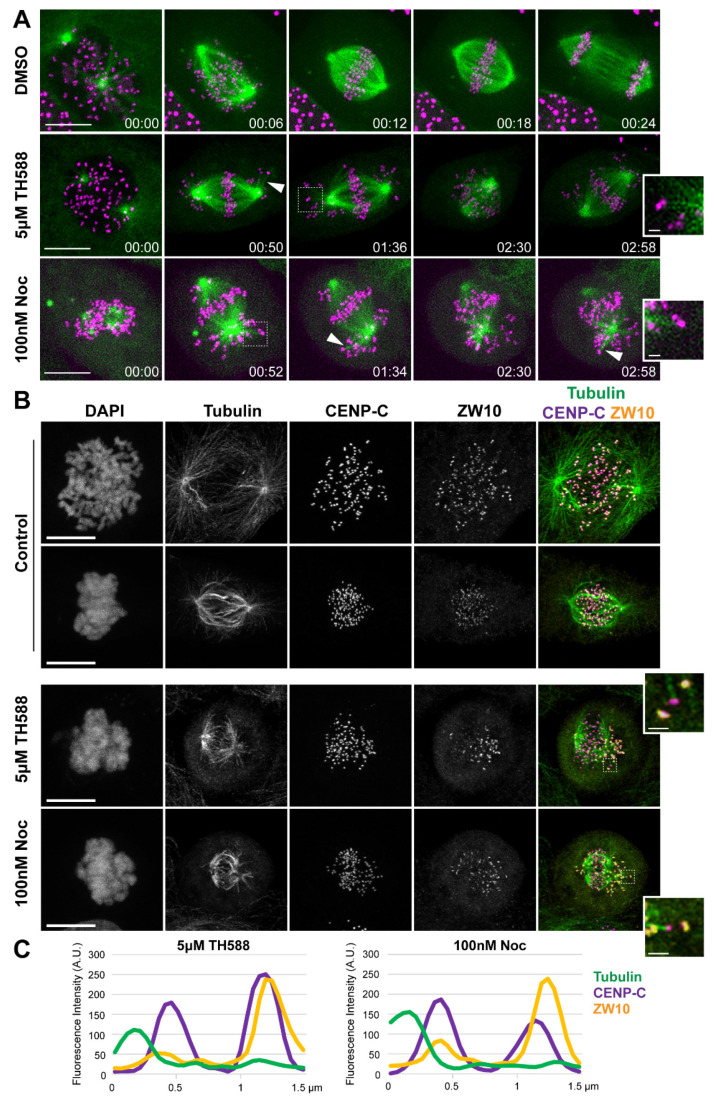
TH588 triggers mitotic arrest and erroneous cell divisions via premature stabilization of kinetochore-MT end-on attachments. (**A**) Representative spinning disk confocal time-series of mitosis in U2OS cells stably expressing CENP-A-GFP (magenta) and mCherry-α-tubulin (green) undergoing indicated treatments. Scale bar 10 µm. Time, hr:min. Arrowheads highlight stabilization of MTs attached to kinetochores. Dashed squares represent regions used for the zoomed insets highlighting syntelic attachments. Inset scale bar 1 µm. (**B**) Representative scanning confocal maximum intensity projections of U2OS cells undergoing indicated treatments, fixed and stained with antibodies against α-tubulin, CENP-C, ZW10, and DAPI to counterstain DNA. CENP-C is shown in magenta, α-tubulin in green and ZW10 in yellow in merged images. Scale bar: 10 µm. Zoomed insets highlight lack of ZW10 in kinetochores attached to MTs. Inset scale bar 1 µm. (**C**) Line profile scans across representative kinetochores showing signal intensity distribution of α-tubulin and ZW10 in relation to the kinetochore marker CENP-C in cells treated with TH588 or nocodazole at indicated concentrations.

Suppression of MT-dynamics interferes with the establishment of correct kinetochore-MT attachments [38]. To examine whether the observed chromosome congression problems, as well as cell divisions with uncongressed chromosomes are a consequence of premature stabilization of end-on kinetochore-MT attachments, we imaged U2OS cells stably expressing GFP-Centromere Protein A (CENP-A) and mCherry-α-tubulin. Whereas DMSO-treated control cells divided normally, the addition of TH588 or low-dose nocodazole prematurely stabilized kinetochore-MTs of uncongressed chromosomes. This eventually led to the formation of erroneous syntelic attachments (having both sister kinetochores attached to the MTs emanating from the same spindle pole), further impairing the chromosome transport toward the metaphase plate (Figure 5A and Figure S3, Videos S9 and S10). To test whether the stable kinetochore-MTs that we observed by imaging U2OS-GFP-CENP-A/mCherry-α-tubulin cells are indeed end-on attached to kinetochores, we immunostained TH588- and low-dose nocodazole-treated U2OS cells against ZW10, a spindle assembly checkpoint (SAC) protein that is removed from kinetochores upon the establishment of stable end-on kinetochore-MT attachment. Since ZW10 was absent from MT-bound kinetochores, this immunostaining confirmed that TH588 and nocodazole induce premature stabilization of MTs associated with the chromosomes surrounding the spindle poles, thereby preventing their congression (Figure 5B,C).

## 4. Discussion

Whereas at high concentrations MTAs either promote or prevent tubulin polymerization, at 10–100-fold lower concentrations they suppress MT dynamics, thereby stabilizing MTs without altering the equilibrium between MT polymer and soluble tubulin [39]. For instance, at high, micromolar concentrations nocodazole prevents tubulin polymerization in vitro [40] and rapidly depolymerizes MTs in cellulo [41]. However, low, nanomolar concentrations of nocodazole reduce MT-growth and shortening rates and increase the time that MTs spend in paused state, both in vitro and in living cells in interphase [36,37]. Since low concentrations of nocodazole also induced mitotic arrest without detectable MT depolymerization [36], these data suggest that the corresponding problems in mitosis result from MT stabilization, rather than depolymerization.

Similar to low-dose nocodazole, TH588 inhibited tubulin polymerization in vitro [15,22], as well as it arrested cells in mitosis and suppressed MT dynamics in interphase [21]. Moreover, the X-ray crystallography-based structural elucidation of the α/β-tubulin: TH588 complex revealed a close overlap in β-tubulin-binding between TH588 and nocodazole [22].

Although these data suggest that mitotic problems induced by either TH588 or low concentrations of nocodazole result from a reduction in spindle MT turnover, it remains unknown whether and how TH588 and low-dose nocodazole affect MT dynamics in mitosis.

Here, we performed a detailed analysis of the impact of TH588 and low-dose nocodazole on MT dynamics within the mitotic spindles of dividing human cancer cells. We show that both of these treatments indeed reduce the MT turnover within mitotic spindle. Interestingly, this suppression of MT dynamics has a destabilizing effect on astral MTs, while it stabilizes interpolar- and kinetochore-MTs. This is consistent with earlier observations that low concentrations of nocodazole selectively disrupts astral MTs, without interfering with the formation of bipolar spindle [42].

Even though TH588 and low-dose nocodazole reduced MT-growth and shortening rates, as well as increased the time that MTs spend in paused state, they also increased the MT catastrophe frequency and decreased the frequency of MT rescue [37]. The increase in MT catastrophe may explain why these drugs destabilize astral MTs, but stabilize interpolar- and kinetochore-MTs. The MT catastrophe rate is suppressed by the tension applied on kinetochores [43,44] and both microneedle-based force application at kinetochores [45,46] and polar-ejection forces (PEFs) exerted on chromosome arms [47,48] stabilize kinetochore-MT attachments. Moreover, kinetochore-based proteins, such as CLASPs, stabilize MT plus-ends by suppressing MT catastrophes and promoting rescue [49,50,51,52,53]. Thus, it is appealing to hypothesize that the increase in catastrophe rate, induced by TH588 and low-dose nocodazole, selectively disrupts astral MTs, while kinetochore-MTs remain protected by the suppressive impact of chromosomes on MT catastrophe rate. In a similar way, the increase in catastrophe rate may be attenuated on interpolar MTs by the actions of MT-crosslinking motors kinesin-5/EG5 [54] and kinesin-12/KIF15 [55], both of which were recently reported to suppress MT catastrophes. With higher concentrations of nocodazole, the increase in MT catastrophes may prevail the MT-stabilizing effects and therefore induce MT depolymerization.

We also show that the spindle MTs-associated stabilizing effect of TH588 and nocodazole results in a premature formation of kinetochore-MT end-on attachments on uncongressed chromosomes, which leads to severe chromosome congression problems and mitotic arrest. At the pole, the uncongressed monotelic chromosomes, with a single kinetochore-MT attachment being stabilized, keep the SAC activated due to the presence of unattached sister kinetochore [56]. Subsequent stabilization of the sister kinetochore-MT attachment leads to the formation of stably attached syntelic chromosomes. These chromosomes cannot congress to the spindle equator most likely due to a lack of activity of dynein and kinesin-7/CENP-E, the two motor proteins required for congression of laterally attached peripheral chromosomes [33,57], as both dynein and CENP-E are largely removed from the end-on attached kinetochores. Eventually, the cells with stably attached syntelic chromosomes satisfy the SAC and divide. Under these circumstances, the SAC satisfaction could occur via intra-kinetochore stretching or structural changes at kinetochores that are triggered by stable end-on attachments [58,59,60].

In addition to stabilizing the spindle MTs, TH588 and low-dose nocodazole prevent the formation of astral MTs that connect mitotic spindle with the cell cortex and are required for proper spindle positioning and orientation of cell division. Thus, these treatments do not only increase the chance of cells dividing with erroneously attached chromosomes, but may also compromise the proper spindle positioning (as observed in Video S9).

Interestingly, whereas the mitotic arrest induced by TH588 or low-dose nocodazole in cancer cells mainly ends in cell death or in cell division with unresolved erroneous kinetochore-MT attachments, in non-tumor cells we observed a significant number of normal cell divisions. This difference is consistent with previous observations that TH588 more efficiently kills cancer cell lines [13]. In addition to its inhibitory effect on MTH1, the selectivity of TH588 toward cancer cells may at least partially rely on its MT-associated activity. For example, non-tumor cells used in this study (RPE-1) are diploid, compared to the two nearly triploid cancer cell lines (U2OS and HeLA). It remains to be addressed by future studies whether, because of having more chromosomes, the polyploid cancer cells are less efficient in correcting prematurely stabilized kinetochore-MT attachments and thereby more prone to cell death upon the treatment with TH588.

## 5. Conclusions

In conclusion, this study demonstrates that the individual actions of two MT-targeting drugs, TH588 and low concentration of nocodazole, reduce MT turnover within the mitotic spindle of dividing cells. This MT-stabilizing effect leads to premature formation of kinetochore-MT end-on attachments on uncongressed chromosomes, which consequently cannot be transported to the cell equator. The resulting mitotic arrest is mainly resolved either by cell death or by cell division with uncongressed chromosomes that may lead to the problems in the following cell cycle. Both of these cell fates could contribute to the selective effect associated with the TH588 activity in cancer cells.

## Data Availability

The data presented in this study are available on request from the corresponding author.

## Supplementary Materials

The following are available online at https://www.mdpi.com/article/10.3390/cancers13235995/s1. Figure S1: TH588 induces severe chromosome congression problems in HeLa cells. Figure S2: TH588 induces severe chromosome congression problems in RPE-1 cells. Figure S3. Low-dose nocodazole-induced premature kinetochore-MT stabilization results in cell division with syntelic attachments. Video S1: Reduced MT dynamics in TH588- and nocodazole-treated interphase cells. Video S2: U2OS cell treated with DMSO undergoing normal mitosis. Video S3: Severe mitotic problems in U2OS cells treated with 5 µM TH588. Video S4: Mitotic problems in U2OS cells treated with 100 nM nocodazole. Video S5: Severe mitotic problems in HeLa cells undergoing TH588 and nocodazole treatments. Video S6: Severe mitotic problems in RPE-1 cells undergoing TH588 and nocodazole treatments. Video S7: Abrogated astral MT dynamics in TH588 and nocodazole treated metaphase-arrested cells. Video S8: Reduced spindle MT turnover upon TH588- and nocodazole-treatments. Video S9: Premature stabilization of kinetochore-MTs upon TH588- and nocodazole-treatments. Video S10: Premature stabilization of kinetochore-MTs in dividing and arrested cells upon low-dose nocodazole treatment.
