# Supplementary material for: TH588 and Low-Dose Nocodazole Impair Chromosome Congression by Suppressing Microtubule Turnover within the Mitotic Spindle
Source: Cancers (Basel). 2021 Nov 29;13(23):5995. doi: 10.3390/cancers13235995 (PMC8657032; doi:10.3390/cancers13235995)
Supplement: Supplementary file 1 [file cancers-13-05995-s001.zip › Rajendraprasad et al_supplement.pdf]

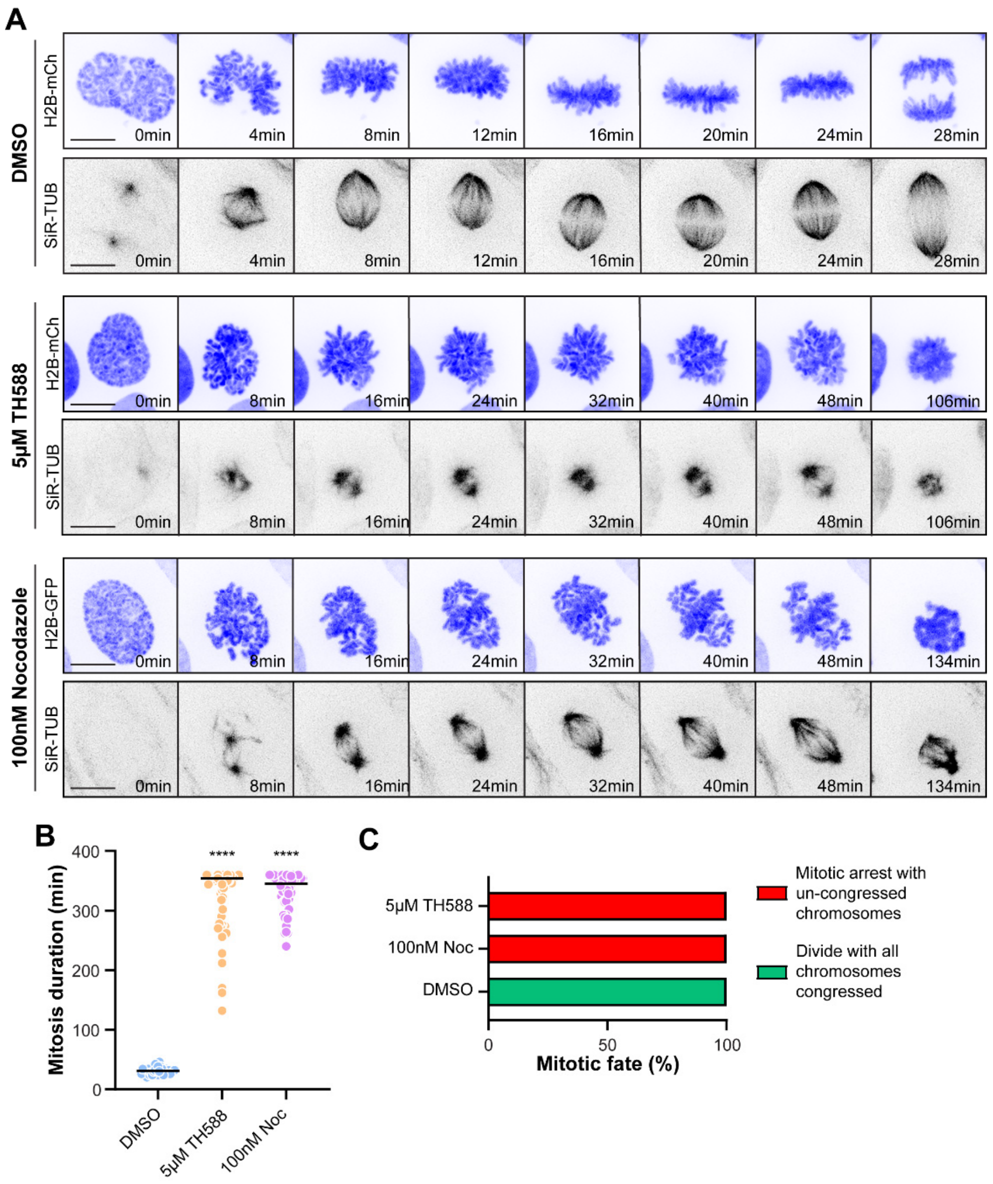

Figure S1. TH588 induces severe chromosome congression problems in HeLa cells. (**A**) Representative spinning-disk confocal time-series of mitosis in HeLa cells stably expressing H2B-mCherry and tubulin stained using SiR-tubulin undergoing indicated treatments. Scale bar 10μm. (**B**) Quantification of duration of mitosis in HeLa cells undrgoing indicated treatments. Scatter plot graphs with median are plotted from 3 independent experiments (n = 30 cells for DMSO, 69 cells for 5μM TH588, and 62 cells for 100nM nocodazole). (**C**) Quantification of the fate of mitotic cells from 3 independent experiments. ****p<0.0001.

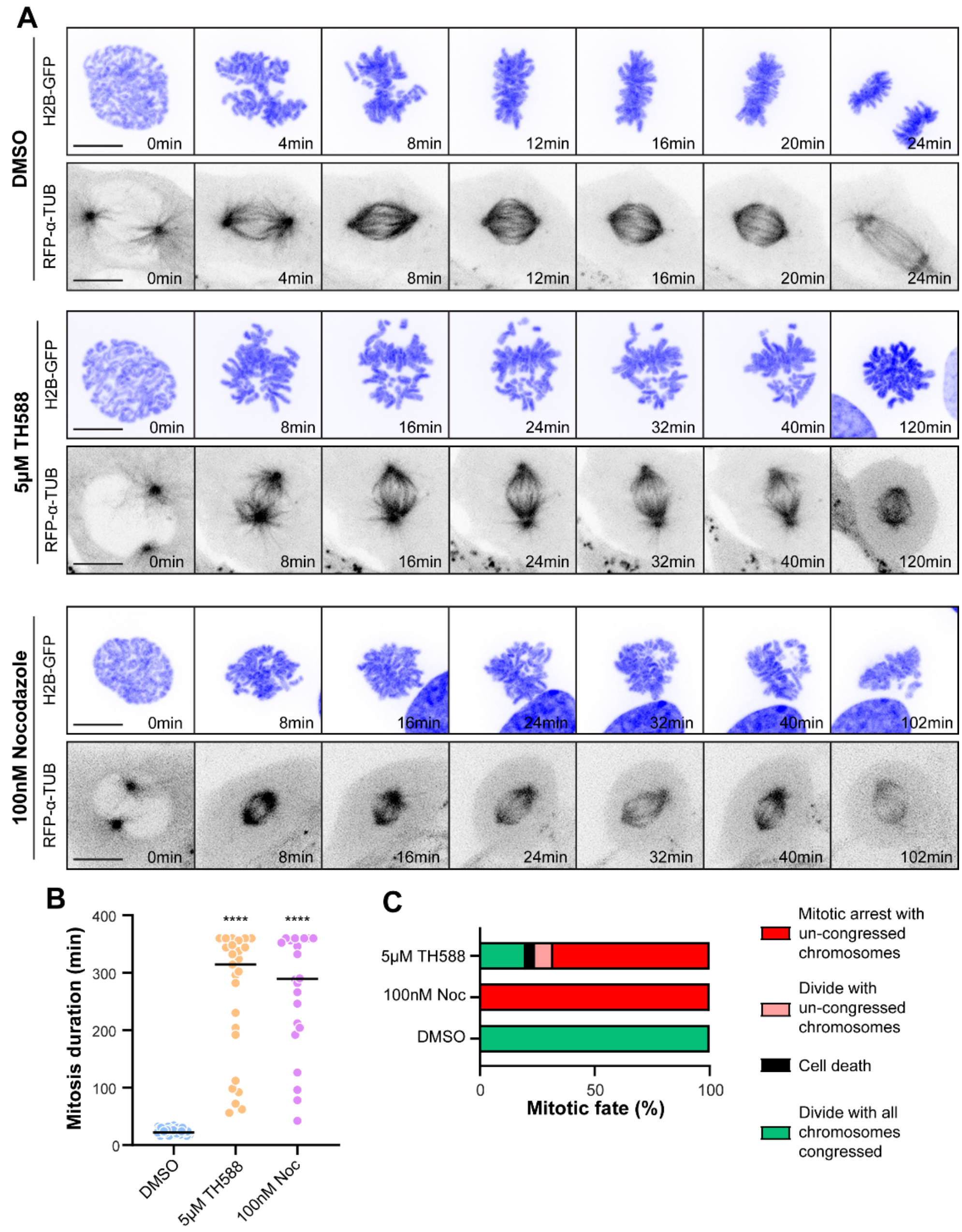

Figure S2. TH588 induces severe chromosome congression problems in RPE-1 cells. (**A**) Representative spinning-disk confocal time-series of mitosis in RPE1 cells stably expressing H2B-GFP/RFP-α-tubulin following indicated treatments. Scale bar 10μm. (**B**) Quantification of duration of mitosis in U2OS cells undergoing indicated treatments. Scatter plot graphs with median are plotted from 3 independent experiments (n = 47 cells for DMSO, 25 cells for 5μM TH588, 22 cells for 100nM nocodazole). (**C**) Quantification of the fate of mitotic cells from 3 independent experiments. ****p<0.0001.

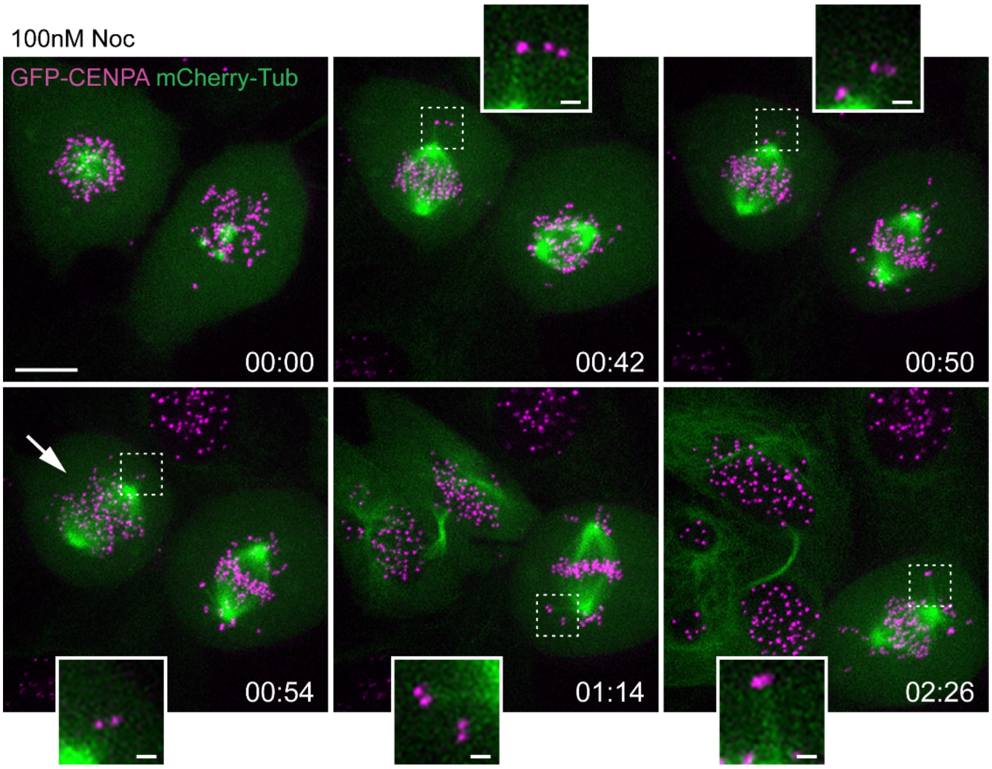

Figure S3. Low-dose nocodazole-induced premature kinetochore-MT stabilization results in cell division with syntelic attachments. Representative spinning disk confocal time-series of mitosis in U2OS cells stably expressing CENP-A-GFP (magenta) and mCherry-α-tubulin (green) upon treatment with 100nM nocodazole. Scale bar 10μm. Time, hr:min. Arrow highlights onset of anaphase. Zoomed insets highlight syntelic attachments. Inset scale bar 1μm.

Supplementary videos legends:

Video S1. Reduced MT dynamics in TH588- and nocodazole- treated interphase cells. MT dynamics in U2OS cells stably expressing EB1-GFP undergoing indicated treatments. Time, s.

Video S2 – 4. Severe mitotic problems in U2OS cells undergoing TH588 and nocodazole treatments. Spinning-disk confocal live-cell imaging of dividing U2OS cells stably expressing H2B-GFP (blue) /mCherry-α-tubulin (grey) undergoing indicated treatments. Time, hour:min.

Video S5. Severe mitotic problems in HeLa cells undergoing TH588 and nocodazole treatments. Spinning-disk confocal live-cell imaging of dividing HeLa cells stably expressing H2B-mCherry (blue) and tubulin stained using SiR-tubulin (grey) undergoing indicated treatments. Time, hour:min.

Video S6. Severe mitotic problems in RPE-1 cells undergoing TH588 and nocodazole treatments. Spinning-disk confocal live-cell imaging of dividing RPE-1 cells stably expressing H2B-GFP (blue) /RFP-α-tubulin (grey) undergoing indicated treatments. Time, hour:min.

Video S7. Abrogated astral MT dynamics in TH588 and nocodazole treated metaphase-arrested cells. MT dynamics in metaphase arrested U2OS cells stably expressing EB1-GFP undergoing indicated treatments. Time, s.

Video S8. Reduced spindle MT turnover upon TH588- and nocodazole- treatments. U2OS cells stably expressing PA-GFP-α-tubulin (cyan) and mCherry-α-tubulin (red) undergoing indicated treatments. 405nm laser-based photoactivation was performed to monitor MT turnover and flux using spinning-disk confocal microscopy. Time, min:s.

Video S9. Premature stabilization of kinetochore-MTs upon TH588- and nocodazole- treatments. Spinning-disk confocal live-cell imaging of dividing U2OS cells stably expressing CENP-A-GFP (magenta) and mCherry α-tubulin (green) undergoing indicated treatments. Time, hour:min.

Video S10. Premature stabilization of kinetochore-MTs in dividing and arrested cells upon low-dose nocodazole- treatment. Spinning-disk confocal live-cell imaging of dividing U2OS cells stably expressing CENP-A-GFP (magenta) and mCherry α-tubulin (green) undergoing indicated treatments. Time, hour:min.
